# Is Generalized and Segmental Dystonia Accompanied by Impairments in the Dopaminergic System?

**DOI:** 10.3389/fneur.2021.751434

**Published:** 2021-11-18

**Authors:** Jun Ikezawa, Fusako Yokochi, Ryoichi Okiyama, Satoko Kumada, Maya Tojima, Tsutomu Kamiyama, Takashi Hanakawa, Hiroshi Matsuda, Fumiaki Tanaka, Yasuhiro Nakata, Eiji Isozaki

**Affiliations:** ^1^Department of Neurology, Tokyo Metropolitan Neurological Hospital, Tokyo, Japan; ^2^Department of Neurology and Stroke Medicine, Yokohama City University Graduate School of Medicine, Yokohama, Japan; ^3^Department of Neuropediatrics, Tokyo Metropolitan Neurological Hospital, Tokyo, Japan; ^4^Department of Neurology, Kyoto University Graduate School of Medicine, Kyoto, Japan; ^5^Integrative Brain Imaging Center, National Center of Neurology and Psychiatry, Tokyo, Japan; ^6^Department of Integrated Neuroanatomy and Neuroimaging, Kyoto University Graduate School of Medicine, Kyoto, Japan; ^7^Department of Neuroradiology, Tokyo Metropolitan Neurological Hospital, Tokyo, Japan

**Keywords:** dystonia, substantia nigra, dopaminergic system, dopamine transporter single photon emission computed tomography, neuromelanin-sensitive MRI, Parkinson's disease

## Abstract

**Background:** The pathogenesis of dystonia is remarkably diverse. Some types of dystonia, such as DYT5 (DYT-*GCH1*) and tardive dystonia, are related to dysfunction of the dopaminergic system. Furthermore, on pathological examination, cell loss in the substantia nigra (SN) of patients with dystonia has been reported, suggesting that impaired dopamine production may be involved in DYT5 and in other types of dystonia.

**Objectives:** To investigate functional dopaminergic impairments, we compared patients with dystonia and those with Parkinson's disease (PD) with normal controls using neuromelanin-sensitive magnetic resonance imaging (NM-MRI) and dopamine transporter single photon emission computed tomography (DAT SPECT).

**Methods:** A total of 18, 18, and 27 patients with generalized or segmental dystonia, patients with PD, and healthy controls, respectively, were examined using NM-MRI. The mean area corresponding to NM in the SN (NM-SN) was blindly quantified. DAT SPECT was performed on 17 and eight patients with dystonia and PD, respectively. The imaging data of DAT SPECT were harmonized with the Japanese database using striatum phantom calibration. These imaging data were compared between patients with dystonia or PD and controls from the Japanese database in 256 healthy volunteers using the calibrated specific binding ratio (cSBR). The symptoms of dystonia were evaluated using the Fahn–Marsden Dystonia Rating Scale (FMDRS), and the correlation between the results of imaging data and FMDRS was examined.

**Results:** The mean areas corresponding to NM in the SN (NM-SN) were 31 ± 4.2, 28 ± 3.8, and 43 ± 3.8 pixels in patients with dystonia, PD, and in healthy controls, respectively. The mean cSBRs were 5 ± 0.2, 2.8 ± 0.2, 9.2 (predictive) in patients with dystonia, PD, and in healthy controls, respectively. The NM-SN area (*r* = −0.49, *p* < 0.05) and the cSBR (*r* = −0.54, *p* < 0.05) were inversely correlated with the FMDRS. There was no significant difference between the dystonia and PD groups regarding NM-SN (*p* = 0.28). In contrast, the cSBR was lower in patients with PD than in those with dystonia (*p* < 0.5 × 10^−6^).

**Conclusions:** Impairments of the dopaminergic system may be involved in developing generalized and segmental dystonia. SN abnormalities in patients with dystonia were supposed to be different from degeneration in PD.

## Introduction

Dystonia is a clinically and etiologically diverse group of disorders. Traditionally, all dystonia cases have been viewed as disorders of the basal ganglia. However, recently, there has been an increasing appreciation for the involvement of other brain regions, including the cerebellum, thalamus, midbrain, and cortex. The concept of dystonia is changing to that of a “network” disorder (1). However, in general, the pathogenesis of dystonia does not involve the substantia nigra (SN). Although the SN is an input system of the basal ganglia, multiple studies using dopamine transporter single photon emission computed tomography (DAT SPECT) have failed to find significant presynaptic dopaminergic impairment in patients with dystonia (2–7).

However, some types of dystonia are caused by reduced functioning of the dopaminergic system, similar to PD. First, DYT5 (DYT-*GCH1*, Segawa disease) is adopa-responsible dystonia (DRD) type caused by guanosine triphosphate cyclohydrolase 1 (GCH-1) deficiency, which is a rate-determining enzyme for dopamine production (8). *GCH-1* is responsible for PD and even in the same DYT5 family; some patients present with DRD, and others present with Parkinsonism (9). DYT5 is not the only dystonia type caused by dysfunction of dopaminergic system. In addition to DYT5, the enzymatic defects in the dopamine biosynthetic pathway also cause dystonia [i.e., 6-pyruvoyltetrahydropterin synthase, sepiapterin reductase, dihydropteridine reductase, tyrosine hydroxylase (TH), and aromatic L-amino acid decarboxylase (AADC)] (10).

Second, dystonia and Parkinson's disease exhibit some clinical overlaps. Dystonia symptoms can be observed in ~20% of patients with PD during wearing-off and early morning (off dystonia) (11). In contrast, dystonia is sometimes accompanied by Parkinsonism (dystonia-Parkinsonism) (12). A combination of dystonia and Parkinsonism may also occur in combined dystonia cases, such as DYT3, DYT12, and DYT13, as well as in heredodegenerative diseases, such as in Wilson's disease, Huntington's disease, and spinocerebellar ataxias. Moreover, dystonia frequently occurs as a presenting symptom in juvenile-onset PD, especially in autosomal recessive genetic Parkinsonism, such as PARK-*PARKIN* (PARK2) and PARK-*SNCA* (PARK1) mutations (13). Third, pharmacological antagonists of dopamine receptors can cause both tardive dystonia and drug-induced Parkinsonism (14, 15).

Recently, it has been reported that the number of pigmented cells in the SN was reduced in 13 patients with dystonia (six with generalized dystonia, seven with segmental or focal dystonia, and seven unknown cases) (15). Moreover, perinuclear inclusion bodies (16) and enlargement of pigmented cells (17) in the SN were observed in patients with DYT1 (DYT-*TORA*). Taken together, it is possible that dopaminergic dysfunction might be involved in DRD and in other types of dystonia.

Therefore, the purpose of this study was to investigate dopaminergic involvement in patients with generalized and segmental dystonia through imaging analysis. We used neuromelanin-sensitive magnetic resonance imaging (NM-MRI) and DAT SPECT (^123^I FP-CIT SPECT). NM-MRI can detect the amount of NM, a by-product of dopamine metabolism, in the SN (18). DAT SPECT visualizes the DAT function in presynaptic dopaminergic nerve terminals and is valuable for the diagnosis and evaluation of PD (19).

## Patients and Methods

This retrospective study was approved by the ethics committee of Tokyo Metropolitan Neurological Hospital (TS-H30-053). Informed consent was obtained using the opt-out method (use from the database for patients with dystonia and those with PD) and in writing (MRI imaging to research for healthy controls). Our protocol followed the tenets of the Declaration of Helsinki.

### Patients and Healthy Controls

Diagnosis and classification of dystonia were confirmed by more than two experienced neurologists at the Tokyo Metropolitan Neurological Hospital between 2017 and 2019, according to the consensus statement of the Movement Disorder Society (MDS) (20). The diagnosis was based on medical history and physical examination and further confirmed by investigation into the phenomenon of muscle co-construction and overflow using surface electromyography. The patients with dystonia in this study were selected retrospectively according to the following criteria: (1) aged between 20 and 60 years and dystonia as the main manifestation; (2) lack of positive imaging findings on MRI screening; (3) lack of or minor cognitive decline; (4) no neurological symptoms other than dystonia had worsened or appeared in the 10 years prior to this study according to the judgement of the patient/caregiver; and (5) no observation of obvious findings suggesting the presence of a neurodegenerative disease.

Eighteen patients with dystonia (sex, 12 males and six females; mean age, 40 ± 2.6 years; mean disease duration, 25 ± 4 years; three, 11, and four cases of inherited, idiopathic, and acquired dystonia, respectively; 16 and two cases of generalized and segmental dystonia, respectively), fulfilled our criteria (Table 1).

**Table 1:** Clinical details of patients.

**Patient**	**Age (years)**	**Sex**	**Disease duration (years)**	**Distribution of dystonia**	**FMDRS total score**	**Etiology**	**Genetical testing**	**Combined other movement disorders**	**Levodopa response**	**Therapy**	**Administration of specific drugs influencing DAT SPECT results**
1	46	F	10	G	13.5	ID	Neg in whole exome analysis	Parkinsonism	Excellent	Drug, GPi-DBS	Nothing
2	25	M	5	G	41	ID	Neg in *TORA*	Parkinsonism	No (only Parkinsonism responded)	GPi-DBS	Nothing
3	51	M	43	G	13	CP (A)	Neg in *TORA*		No	GPi-DBS	Nothing
4	52	F	43	S	17	DYT11 (IN)	A Mutation in *SGCE*	myoclonus	No	GPi-DBS	Nothing
5	45	M	6	S	16	ID	NA		No	GPi-DBS	Nothing
6	44	M	43	G	9	CP (A)	NA		No	Drug	Nothing
7	26	M	2	G	12	TD (A)	NA		No	BTX	Nothing
8	52	F	32	G	31	ID	NA		No	GPi-DBS	Nothing
9	21	M	18	G	10	DYT1 (IN)	CAG Del in *TORA*		No	GPi-DBS	Nothing
10	31	M	18	G	14	ID	Neg in *TORA*		No	BTX	Nothing
11	34	M	33	G	21.5	ID	Neg in *TORA*		No	GPi-DBS	Nothing
12	29	M	23	G	8	ID	Neg in *GCH1*		Excellent	Drug	Nothing
13	52	F	42	G	22.5	ID	Neg in whole exome analysis		No	GPi-DBS	Milnacipran hydrochloride 150 mg
14	57	M	56	G	16.5	CP (A)	NA		No	Drug	Mianserin hydrochloride 10 mg
15	32	F	10	G	14	ID	Neg in whole exome analysis		No	Drug	Nothing
16	45	M	31	G	12	DYT11 (IN)	A Mutation in *SGCE*	myoclonus	No	GPI-DBS	Escitalopram oxalate 10 mg
17	41	F	17	G	10	ID	Neg in whole exome analysis		Partial	Drug, GPi-DBS	Nothing
18	49	M	33	G	13	ID	NA		No	BTX	Nothing
Dystonia group Av (±SEM)	40 (±2.6)	M 12, F 6	25 (±4.0)	G 16/S 2	16.3				Effective 3, None 15		
PD group Av (±SEM)	52 (±1.2)	M 12, F 6	2.1 (±0.2)								
Control group Av (±SEM)	39 (±1.8)	M 15, F 12									

*Av, average; PD, Parkinson's disease; SD, standard deviation; M, male; F, female; G, generalized; S, segmental; FMDRS, Fahn-Marsden Dystonia Rating Scale; IN, inherited; A, acquired; ID, idiopathic; CP, cerebral palsy; TD, tardive dystonia; Neg, negative; NA, not applicable; SGCE, ε-sarcoglycan; TORA, torsinA; GCH-1, guanosine triphosphate cyclohydrolase 1; GPi, internal segment of the globus pallidus; DBS, deep brain stimulation; SEM, standard error of the mean*.

The symptoms of dystonia were evaluated using the Fahn–Marsden Dystonia Rating Scale (FMDRS) (21). NM-MRI, DAT SPECT, and FMDRS evaluations were all performed within 1 month for all cases. Two patients with opisthotonus as the main symptom had very mild rigidity. Two patients with segmental dystonia presented with dystonia symptoms in the lower cranial region, the cervical region, and the upper limbs, but no dystonia symptoms in the trunk. All patients had been administered levodopa, which was effective for dystonia in three patients. Eleven patients with dystonia were treated with pallidal deep brain stimulation after participation in this study.

The patients in the PD group were also selected retrospectively if they met the following inclusion criteria: (1) age of 20–60 years (to match the age of those in the dystonia group) with sporadic PD; (2) fulfillment of the MDS diagnosis criteria (22); (3) within 5 years from the onset of motor symptoms; (4) absence of motor fluctuation, such as wearing-off or dyskinesia; and (5) Hoehn and Yahr Stage 1 or 2 with no treatment or after an overnight washout of the dopaminergic drugs. In total, 18 patients with PD (sex, 12 male and six female individuals; age, 52 ± 1.2 years) met these criteria (Table 1).

The healthy control group comprised age-matched 27 healthy volunteers (sex, 15 male and 12 female individuals; age, 39 ± 1.8 years) with no medical history of neurological disease, who underwent NM-MRI prospectively.

### Imaging Analysis

#### NM-MRI

Neuromelanin-sensitive magnetic resonance imaging was performed in all groups (dystonia, PD, and control groups) to evaluate the deposition of NM in the SN. The patients underwent 3T MRI (Discovery MR750; GE Healthcare, Chicago, IL, USA). After the initial localization settings, the optimized 2D T1-weighted images (T1-WI) were acquired with the following parameters: TR, 600 ms; TE, 14 ms; flip angle, 15; slice thickness, 2.5 mm; slices 11; field of view, 18; matrix size, 446 × 256; scanning time, 5.24 min; magnetization transfer pulse. To minimize the partial volume effect with tissues other than the SN, the slices were oriented perpendicular to the brain stem and tilted at 20° to a transaxial plane through the anterior-posterior commissure line (23, 24).

The NM-SN positive area, which was the high-intensity area of NM-MRI corresponding to NM in the SN, was blindly quantified using the processing software, Image J (NIH, Bethesda, MD, USA). In brief, as described in previous reports (24, 25), the concept was to remove the background noise except for the SN. The 2–3 slices containing the SN were chosen because of the identification of NM as a T1-weighted high-intensity region on NM-MRI. The image slices were converted to eight-bit grayscale images. The threshold was set by a blinded investigator at the level where the noise contrast in the midbrain disappeared, leaving high-signal-intensity areas on T1-WI in the SN. After setting the threshold, the pixels in the SN were automatically calculated, yielding the value of the MN-SN area.

#### DAT SPECT

Dopamine transporter single photon emission computed tomography was also performed in 17 patients with dystonia and eight patients with PD to confirm dopaminergic impairments from another side. The patients received an intravenous injection of 167 MBq of ^123^I FP-CIT. After 4 h, SPECT images of the head were acquired for 30 min in a 128- × -128 matrix of 3.3-mm pixels using a dual-head gamma camera with low-energy and high-resolution collimators (Discovery NM630; GE Healthcare). On DAT SPECT, SPECT images were reconstructed with the iterative algorithm. Reconstructed data were quantified using DaTView (Nihon Medi-Physics, Tokyo, Japan) based on the Southampton method (26). The specific binding ratios (SBR) were calculated as follows:


$$\begin{array}{l} \text{SBR}=\left(\text{uptake of the striatum }-\text{uptake of the whole brain}\right)/\\ \text{ uptake of the whole brain}. \end{array}$$

In this method, geometrical volumes of interest (VOIs) larger than the striatum were used to consider partial volume effects (Supplementary Figure 1). Radiological technicians were not allowed to manually define the striatum, and their interventions were limited to the shifting of template geometrical VOIs. The Southampton method is described as highly reproducible, with an operator-introduced variability of only 4% (26).

SBR values decrease linearly with aging (27–29). Thus, we compared the SBR values of patients with those in the database of Japanese healthy controls (*n* = 256) (27). As the striatum phantoms (Nihon Medi-Physics) were filled with different densities of ^123^I, a calibration curve was prepared. The reference database was harmonized using striatum phantom calibration. The calibration curve was also arranged by acquiring a striatum phantom at the Tokyo Metropolitan Neurological Hospital (y = 1.40496 x + 0.20537, *r* = 0.99852). The calibrated SBR (cSBR) was calculated at Tokyo Metropolitan Neurological Hospital and compared with those in the database as controls. As an indicator of laterality, the absolute values of the asymmetry index (AI) were calculated as follows: AI = 200 (R–L)/(R+L) (30).

R and L represent the right and left striatal cSBRs, respectively.

### Statistical Analysis

Statistical analysis was performed using Student's *t*-test, the Spearman rank correlation coefficient, and the Pearson correlation coefficient according to data properties. The level of significance was set at *p* < 0.05. Statistical analyses were performed with IBM SPSS Statistics software version 24 (IBM Corp., Armonk, NY, USA).

## Results

**Figure 1:**
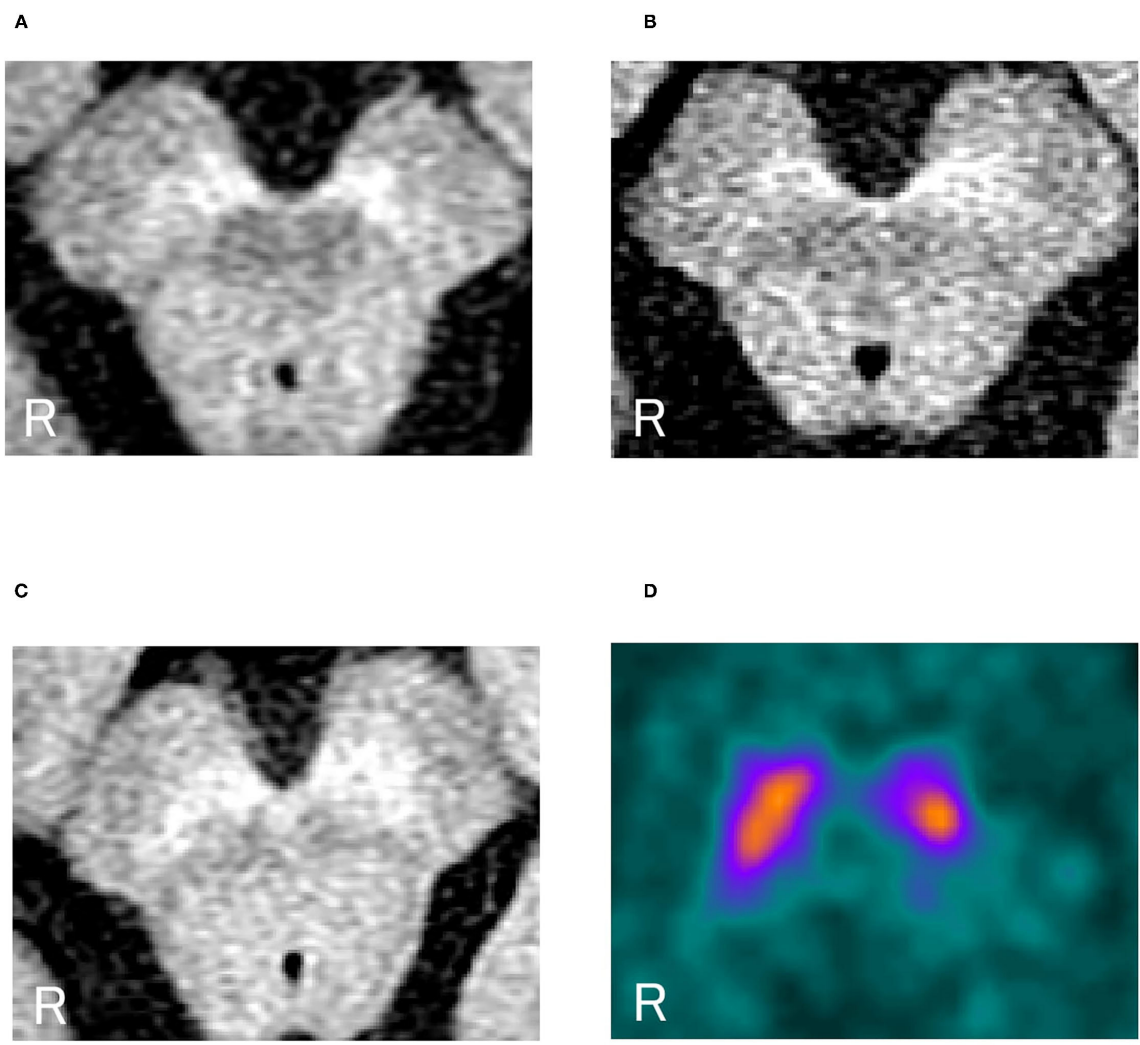
**(A)** Neuromelanin-sensitive magnetic resonance imaging in a representative dystonia case (a patient with DYT-*SGCE*). **(B)** NM-MRI in a representative PD case. **(C)** NM-MRI in a representative control case. **(D)** DAT SPECT in a representative case of a female patient with dystonia (DYT-*SGCE*). Her cSBR was 4.79/3.33. Her age-estimated cSBR was 8.40. The 95% lower limit of prediction intervals on the cSBR for 50–59 years was 5.95. NM-MRI, neuromelanin magnetic resonance imaging; DYT-*SGCE*, dystonia*-epsilon sarcoglycan* gene; PD, Parkinson's disease; DAT SPECT, dopamine transporter single-photon emission contrast tomography; cSBR, calibrated specific binding ratio.

Figure 1 shows representative images. A representative case of dystonia was of a 53-year-old female patient with childhood-onset myoclonus-dystonia who did not receive any oral medication. She was genetically diagnosed with DYT-*SGCE* (DYT11). Understandably, she did not show Parkinsonism. Nevertheless, the high-intensity area in the midbrain on NM-MRI reflecting NM pigmentation was smaller than that of controls, especially in the ventrolateral SN, as observed in PD cases (Figures 1A–C). In DAT SPECT, the cSBR was 4.79/3.33. Her age-estimated cSBR from the database (27) was 8.40, and the 95% lower limit of prediction intervals on cSBR for patients aged 50–59 years was 5.95 (Figure 1D). The results of the other individual cases are available in Supplementary Table 1.

**Figure 2:**
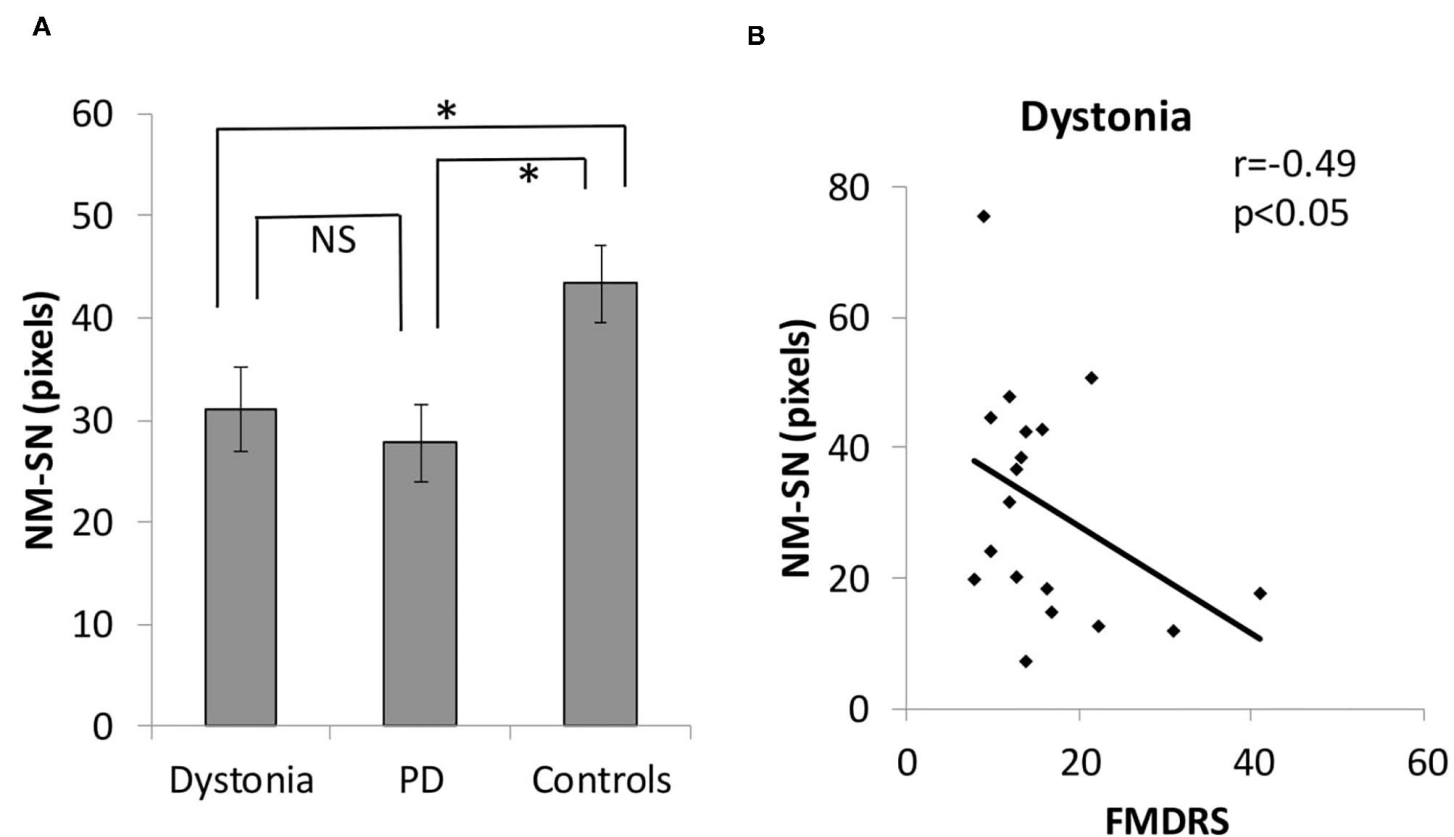
**(A)** Comparison of the positive NM-SN area in patients with dystonia, patients with PD, and healthy controls. **(B)** Correlation between the FMDRS and the NM-SN area in patients with dystonia. Error bars indicate SEM. *Statistically significant difference (*p* < 0.05). NM-SN, neuromelanin-substantia nigra; FMDRS, Fahn-Marsden Dystonia Rating Scale; NS, non-significant; PD, Parkinson's disease; SEM, standard error of the mean.

**Figure 3:**
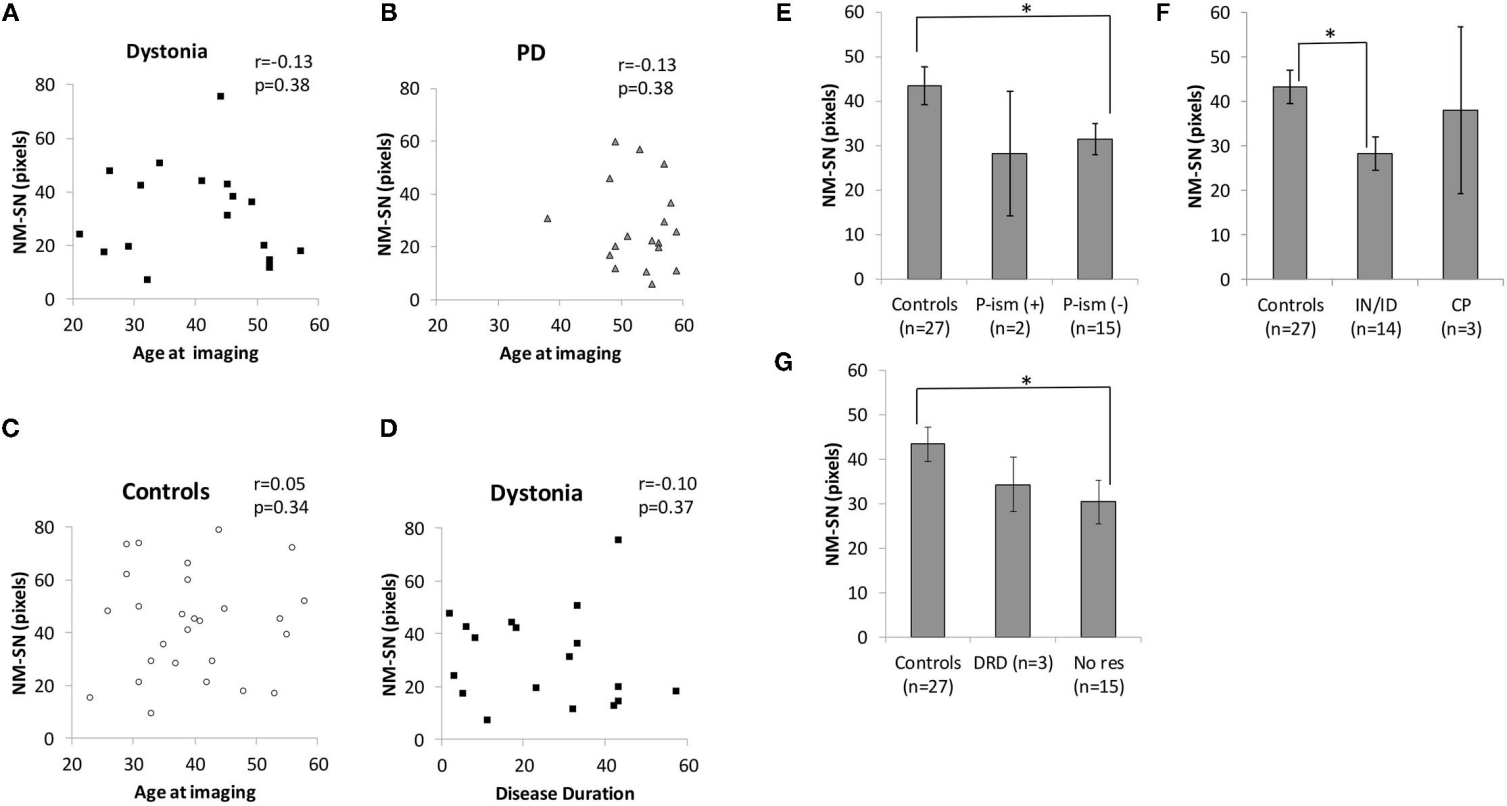
**(A)** Correlation between age at imaging and the NM-SN area in the dystonia group. **(B)** Correlation between age at imaging and the NM-SN area in the PD group. **(C)** Correlation between age at imaging and the NM-SN area in the control group. **(D)** Correlation between disease duration and the NM-SN area in the dystonia group. **(E)** Comparison of the NM-SN area with the presence of Parkinsonism in the dystonia group. P-ism (+) refers to patients with dystonia combined with Parkinsonism (*n* = 2), and P-ism (-) refers to patients with dystonia without Parkinsonism (*n* = 15). **(F)** Comparison of etiology of the NM-SN area. IN/ID means inherited and idiopathic dystonia patients (*n* = 14), and CP refers to cerebral palsy (*n* = 3). **(G)** Comparison of the NM-SN area regarding the responsivity of levodopa. DRD refers to dopa-responsive dystonia (*n* = 3), and No res refers to no responders to levodopa (*n* = 15). Error bars indicate SEM. *Statistically significant difference (*p* < 0.05). NM-SN, neuromelanin-substantia nigra; DRD, dopa-responsive dystonia; PD, Parkinson's disease; CP, cerebral palsy; SEM, standard error of the mean.

Figure 2 shows the overall results of NM-MRI. The NM-SN area of patients with dystonia was significantly smaller than that of controls, but there was no significant difference between patients with dystonia and those with early-stage PD (Figure 2A). Importantly, the NM-SN area was significantly inversely correlated with the FMDRS in the Spearman rank correlation analysis (Figure 2B). In addition, the laterality of the dominant symptomatic side and reduction of NM-SN area coincided in 56% (10/18) of patients with dystonia, which was comparable to 61% (11/18) of those with PD (Supplementary Table 2). Figure 3 shows the results of the NM-MRI subgroup analysis. There was no correlation between the NM-SN area and age in patients with dystonia, those with PD, or in controls (Figures 3A–C). There was also no correlation between the NM-SN area and disease duration in dystonia cases (Figure 3D). The patients with dystonia without Parkinsonism presented a statistically significant decrease in the NM-SN area compared to the controls (Figure 3E). Concerning the dystonia etiologies, the NM-SN area in inherited and idiopathic dystonia cases decreased (Figure 3F). Regarding the relationship between the NM-SN area and levodopa response, the NM-SN area was reduced in patients with dystonia without dopa responsiveness compared to the controls (Figure 3G).

**Figure 4:**
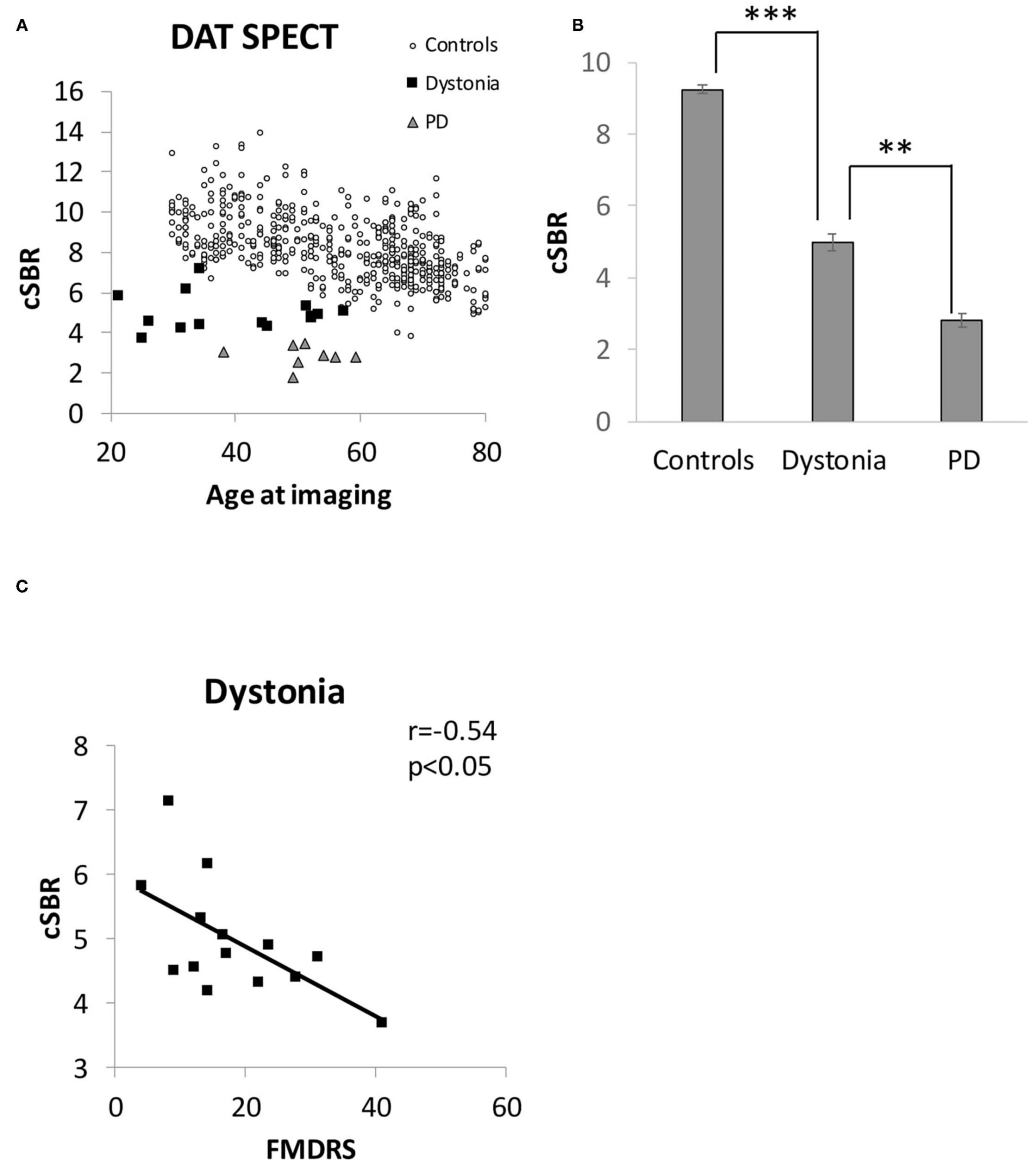
**(A)** Distribution of the cSBR in patients with dystonia, patients with PD, and healthy controls. The control group was obtained from a database of Japanese healthy volunteers (*n* = 256). **(B)** Comparison of the cSBR between patients with dystonia, patients with PD, and healthy controls. The participants in the control group were extracted from the Japanese database to match the mean age of the dystonia group (*n* = 89, 30–50 years) for comparison with that group. **(C)** Correlation between the FMDRS and the cSBR of patients with dystonia. Error bars indicate SEM. **Statistically significant difference (*p* < 0.5 x 10^−6^). ***Statistically significant difference (*p* < 0.5 x 10^−22^). cSBR, calibrated specific binding ratio; FMDRS, Fahn-Marsden Dystonia Rating Scale; PD, Parkinson's disease; SEM, standard error of the mean.

**Figure 5:**
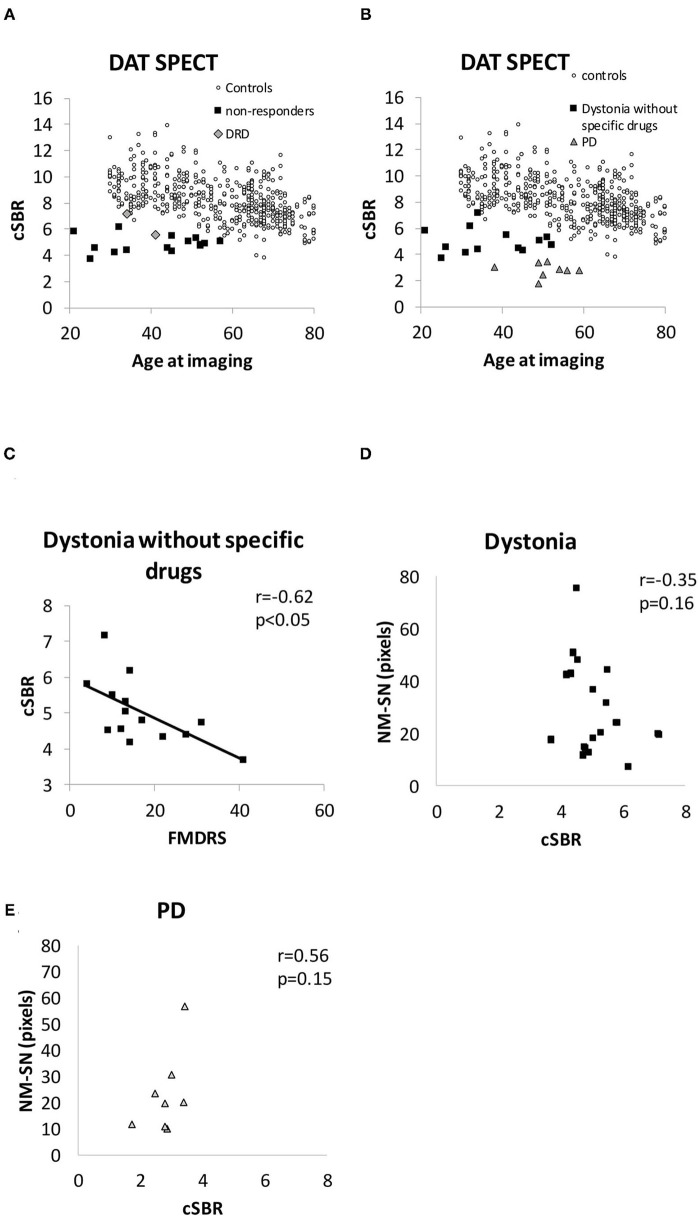
**(A)** Distribution of the cSBR in patients with DRD and non-responders. **(B)** Distribution of the cSBR in patients with dystonia, not taking specific drugs influencing the results of DAT SPECT. **(C)** Correlation between the FMDRS and the cSBR obtained from patients with dystonia not receiving specific drugs. **(D)** Comparison between the cSBR and the NM-SN area in patients with dystonia. **(E)** Comparison between the cSBR and the NM-SN area in patients with PD. cSBR, calibrated specific binding ratio; DAT SPECT, dopamine transporter single-photon emission computed tomography; DRD, dopa-responsive dystonia; NM-SN, neuromelanin-substantia nigra; PD, Parkinson's disease; SEM, standard error of the mean.

Figure 4 shows the results of DAT SPECT imaging. All cSBRs in patients aged ≥30 years were lower than the 95% confidence interval of healthy controls (Figure 4A). Importantly, the cSBR in dystonia was significantly higher than that in early-stage PD cases (Figures 4A,B), while the NM-SN area in dystonia and PD cases was comparable. The cSBR was correlated with the FMDRS (Figure 4C), similar to the NM-SN area (Figure 2B). The cSBR in healthy controls obtained from the database (27) was linearly correlated with age, but not in patients with dystonia or PD (Figure 4A). Figure 5 shows the results of the DAT SPECT subgroup analysis. The cSBR in non-responders to levodopa was lower than that in controls (Figure 5A). DAT SPECT results are known to be influenced by specific drugs, such as cocaine, amphetamines, methylphenidate, and selective serotonin reuptake inhibitors (SSRI) (30). We included an analysis performed in patients with dystonia not taking specific drugs (Figure 5B) because three patients with dystonia took SSRIs (Table 1). The correlation between the cSBR and the FMDRS was maintained in 14 patients without these drugs (Figure 5C). The correlation between the NM-SN area and the cSBR was not significant in patients with dystonia (Figure 5D) or in those with PD (Figure 5E).

## Discussion

In this study, we demonstrated impairments in the dopaminergic system in patients with generalized and segmental dystonia using NM-MRI and DAT SPECT, indicating that impaired dopamine production may be related to the pathogenesis of generalized and segmental dystonia. Interestingly, the abnormalities of dopaminergic system observed in patients with dystonia differed from the corresponding of those with PD. This study provides new evidence concerning the dopaminergic mechanisms as network disturbances involved in generalized and segmental dystonia.

Our study demonstrated a clear relationship between dopaminergic impairments and dystonia contrary to the conventional wisdom that SN was not abnormal in dystonia. Although this is the first report using NM-MRI, it was previously reported that patients with dystonia had similar uptake levels as the controls on DAT SPECT examination (15–20). However, the previous reports were not conclusive in three points. First, the participants in relatively reliable studies were limited to cervical dystonia, which is one of the most common types (2, 3). The background of generalized and segmental dystonia is different from that of cervical dystonia. Patients with generalized and segmental dystonia tend to manifest symptoms earlier, and its pathogenesis is much more related to genetics than cervical dystonia (20). Thus, whether the results of cervical dystonia could apply to generalized and segmental dystonia remains unclear. Second, in the studies restricted to DRD or inherited dystonia caused by a single gene, such as DYT1, manifesting mainly in generalized dystonia, the included patients were limited because of the disease rarity [dystonia classification/number of patients; DRD/*n* = 8 (4), DRD/*n* = 2 (5), DYT1/*n* = 1 (6), DYT11/*n* = 1 (7)]. Third, a normal value for each age was not set despite the fact that DAT normal levels decrease with aging (27–29). A significant effect of age was found in the normal database, with an SBR decline rate of 6.3% per decade (27). In cases where the age of the patients varies, a simple comparison of the dystonia patient group with the control group would not be appropriate. Bias because of age variation can be added, and the range of normal values can be very large. In addition, the age of controls was more than two times that of patients with DRD (4), and DAT SPECT examinations were based only on visual evaluation (6, 7). Mild impairments could not be observed in these previous studies. Although molecular imaging studies using DAT SPECT and positron emission tomography failed to find significant presynaptic dopaminergic impairment in DRD cases (31), it had similar limitations (i.e., the small sample size and the insufficient control group). Our results were obtained from a sufficient sample of patients with generalized and segmental dystonia, and, by comparison with the largest database ever reported (27), we succeeded in detecting the DAT impairments in patients with dystonia, which could not be detected to date. Interestingly, these impairments were milder than those in early-stage PD.

We found that the NM-SN area (measured by NM-MRI) and the cSBR (measured by DAT SPECT) in generalized and segmental dystonia cases were correlated with the FMDRS. Similarly, the NM-SN area and the SBR in PD cases are known to be correlated with disease severity, as demonstrated by the Hoehn and Yahr grade, unified Parkinson disease rating scale Part III, and appearance of motor fluctuation (32, 33). However, the NM-SN area in progressive supranuclear palsy (PSP) is not correlated with disease severity (34). This difference dovetails with the popular concepts that Parkinsonism in patients with PD is caused by the loss of dopamine neurons in the SN and that Parkinsonism in PSP cases is mainly attributed to basal ganglia impairments. Thus, the correlation between dopaminergic impairment and disease severity observed in patients with generalized and segmental dystonia supported the possibility that a dysfunction in the dopamine system was involved in the pathogenesis. In addition, the correspondence between NM-SN laterality and symptom laterality in patients with generalized and segmental dystonia was the same as that observed in PD cases.

In contrast, the NM-SN area was comparable to that observed in patients with dystonia and PD, but the cSBR was significantly lower in PD cases. The NM-SN area reflects the amount of NM (18), while the cSBR reflects DAT function in presynaptic dopaminergic nerve terminals (19). Although we could not determine the implicated mechanisms and alterations in the SN of patients with dystonia in this study, the deviation between the NM-SN area and the cSBR suggested that the SN abnormalities observed in dystonia cases may differ from the degeneration observed in PD. We also speculate that the abnormalities in the dopaminergic system were distinct between dystonia and PD cases. Previous works using DYT5-mouse models have shown that the involvement of the nigrostriatal system differed from that observed in PD models of the striatum striosome-matrix pattern of dopamine loss (35) and those with induction of functional alterations in dopamine receptors (36). These findings suggested that dystonia and PD may differ in terms of dopaminergic system involvement.

However, impairments of the dopaminergic system are not considered universal abnormalities in dystonia because its pathogenesis is extremely diverse. In previous reports, patients with DYT1 manifesting generalized dystonia presented with hypertrophy and inclusion bodies in the SN neurons (16, 17), and a pathological reduction of the melanin pigment in DRD was reported in patients with DYT5 (37, 38). Conversely, there was no neuronal inclusion in idiopathic adult-onset idiopathic focal dystonia (39). The studies on DAT SPECT showed normal uptake in cervical dystonia (3, 4). One transcranial sonography study of the SN revealed the existence of hyperechography in DRD (40) and DYT6 (41) that was similar to that observed in PD cases. However, another study reported no sonographic alterations in cervical dystonia and blepharospasm (42). Taken together, the studies on generalized and segmental dystonia, including our study, showed abnormality of the SN and no change in focal dystonia, including cervical dystonia and blepharospasm. Considering that the background of generalized and segmental dystonia is different from that of focal dystonia, the involvement of the dopaminergic system may differ according to the type of dystonia. The onset of generalized and segmental dystonia tends to be earlier, much more related to genetics, and less related to repetitive motions that are often performed by professionals, such as musicians, hairdressers, and sports players, and linked to focal task-specific dystonia (20). Further studies are necessary to compare dopaminergic abnormalities according to various types of dystonia.

Although dopaminergic impairments in dystonia were obvious, only three of 18 cases responded to levodopa. However, it could not be concluded that the decrease in the dopamine levels in generalized and segmental dystonia was unrelated to the pathogenesis because dopaminergic impairments were obvious in our dystonia cohort. Indeed, DYT5 symptoms are recovered by a small amount of levodopa, but the patients with TH deficiency and AADC deficiency are less responsive to dopamine replacement therapy (10). In AADC deficiency, dopamine agonist therapy provided the patients with few benefits, but, after *AADC* gene transfer, the patients showed marked improvement in their dystonia symptoms (43). It is possible that the simple administration of a dopaminergic drug may not be sufficient, and it may be necessary to address other factors, such as the rhythm of dopamine release, to improve dystonia. In addition, drug-induced Parkinsonism is gradually improved by discontinuation of the antipsychotic drug, but tardive dystonia lasts for a long time (13, 14). In general, dopamine replacement therapy in dystonia caused by lack of dopamine is not as effective as it is for PD. Many studies using PET imaging and mouse models suggested that dysfunction of dopamine D2 receptors underlies the pathophysiology of inherited dystonia, which mostly presents with generalized dystonia (44, 45). The chronic dopamine deficiency revealed in this study is supposed to have some effect on D2 receptors.

We acknowledge the heterogeneity of the dystonia group and the small sample size of each category as limitations, despite the fact that dystonia is a diverse disease. In particular, although this is a study on the relationship between dopamine and dystonia, there were only three cases of DRD in this study on NM-MRI and two on DAT SPECT. In addition, the correlation between the NM-SN area and FMDRS is weak (Figure 2B). However, although the evidence is weak, the reduction in the NM-SN area in patients with dystonia seems to be independent of the presence of Parkinsonism (Figure 3E). The NM-SN area and cSBR appeared to be reduced in patients with dystonia with and without a dopa response compared to controls (Figures 3G, 5A). As aforementioned, previous studies on DRD or inherited dystonia caused by a single gene could not find significant differences because of their small scales. Considering our findings, further studies with big sample sizes and an adequate number of healthy controls to detect mild abnormalities are needed to reexamine DAT SPECT in DRD or patients with inherited dystonia.

Another limitation was the fact that this was a retrospective study, and, therefore, DAT SPECT could not be performed in all patients with dystonia and PD. However, although the rate of DAT SPECT in patients with PD was low (8/18), we could perform both imaging studies in 17/18 patients in the dystonia group, which was a sufficient number to demonstrate a relationship between NM-MRI and DAT SPECT. Moreover, the NM-MRI and DAT SPECT data for the healthy control group differed from the measured data and the database, respectively.

This study was also partly limited by the significant difference in age between the dystonia group and PD. Indeed, dopamine function in healthy people changes with age, but the correlation between age and dopamine function is known to disappear in PD (33). In accordance with this finding, there was no correlation between age and the NM-SN area or age and the cSBR among patients with PD in this study. Similarly, neither the NM-SN area nor the cSBR was correlated with age in patients with dystonia. Considering relatively age-stable dopamine function in pathological states including PD and dystonia, we inferred that age differences did not appreciably contribute to the differential imaging results between these two disorders. As a limitation for matching of the disease onset, the peak age of the onset in generalized and segmental dystonia differed from that in PD, and the majority of juvenile-onset PD cases could not be included in this study because these patients often show dystonia symptoms (11, 12). Moreover, it was nearly impossible to compare various severities of dystonia because young and middle-aged patients with severe or moderate dystonia who had received deep brain stimulation therapy were not included in this study as they could not undergo NM-MRI (3T-MRI).

In conclusion, our results suggested dopaminergic involvements in dystonia contrary to conventional wisdom that SN in dystonia cases was unrelated to pathogenesis. The dopaminergic system may be involved as a part of the network disturbance in addition to the basal ganglia, cerebellum, thalamus, and cortex in the generalized and segmental dystonia. The mechanism of SN abnormalities among patients with dystonia was considered to differ from degeneration in patients with PD.

## Data Availability Statement

The raw data supporting the conclusions of this article will be made available by the authors, without undue reservation.

## Ethics Statement

The studies involving human participants were reviewed and approved by the Ethics Committee of Tokyo Metropolitan Neurological Hospital. Written informed consent for participation was not required for this study in accordance with the national legislation and the institutional requirements. Written informed consent was not obtained from the individual(s) for the publication of any potentially identifiable images or data included in this article.

## Author Contributions

JI: conception, design, and first draft. JI and MT: data analysis. JI, TH, HM, and YN: methodology. JI and FY: interpretation. JI, FY, RO, SK, MT, TK, and YN: data collection. FY, RO, SK, MT, TH, HM, FT, YN, and EI: revision. FY and EI: supervision. All the authors approved the final version.

## Funding

TH receives funding of The Brain Mapping by Integrated Neurotechnologies for Disease Studies (Brain/MINDS, 19dm0207070s0101) and Strategic International Brain Science Research Promotion Program (Brain/MINDS beyond, 19dm0307003h0002) from Japan Agency for Medical Research and Development (AMED), KAKENHI grants (19H03536 and 18H04960) from Japan Society for the Promotion of Science (JSPS), and the Intramural Research Grant (30-4) for Neurological and Psychiatric Disorders of the National Center of Neurology and Psychiatry, Japan. HM was supported by Intramural Research Grant (30-3, 30-10) for Neurological and Psychiatric Disorders of the National Center of Neurology and Psychiatry, Japan. FT was supported by Grants-in-Aid for Scientific Research from the Ministry of Education, Culture, Sports, Science, and Technology (#18K07532, #20K11160, #20K07761, #19K07845, #19H03577, #18K07531, #18K07504, and #18K07503), Health and Labor Sciences Research Grant from the Ministry of Health, Labor, and Welfare, Japan (#201711060A), and Grant for Strategic Research Promotion from Yokohama City University (#SK2804).

## Conflict of Interest

Unrelated to this study, FY receives grants from InSightec, Ltd. for a trial on focused ultrasound for Parkinson's disease. The remaining authors declare that the research was conducted in the absence of any commercial or financial relationships that could be construed as a potential conflict of interest.

## Publisher's Note

All claims expressed in this article are solely those of the authors and do not necessarily represent those of their affiliated organizations, or those of the publisher, the editors and the reviewers. Any product that may be evaluated in this article, or claim that may be made by its manufacturer, is not guaranteed or endorsed by the publisher.

## Supplementary Material

Supplementary Figure 1 A process of geometric VOI determination by the Southampton method in representative patients with dystonia. Purple pentagons represent preformed striatum ROI. The radiological technicians were allowed only to shift VOIs in the anterior–posterior and lateral directions. The yellow dotted line represents the center of the right striatum, and the yellow line represents the upper and lower limits of the striatum automatically identified by the software. The white dotted line represents the automatically determined whole brain, and the white line represents a reference ROI 20 mm inward from the white dotted line. VOI, volume of interest; ROI, region of interest.

Supplementary Table 1 Results of NM-MRI and DAT SPECT.

Supplementary Table 2 Correspondence of laterality between the dominant symptomatic side and the other side in imaging.
