# Supplementary figures and images for: Is Generalized and Segmental Dystonia Accompanied by Impairments in the Dopaminergic System?
Source: Front Neurol. 2021 Nov 18;12:751434. doi: 10.3389/fneur.2021.751434 (PMC8638468; doi:10.3389/fneur.2021.751434)

## Slide 1
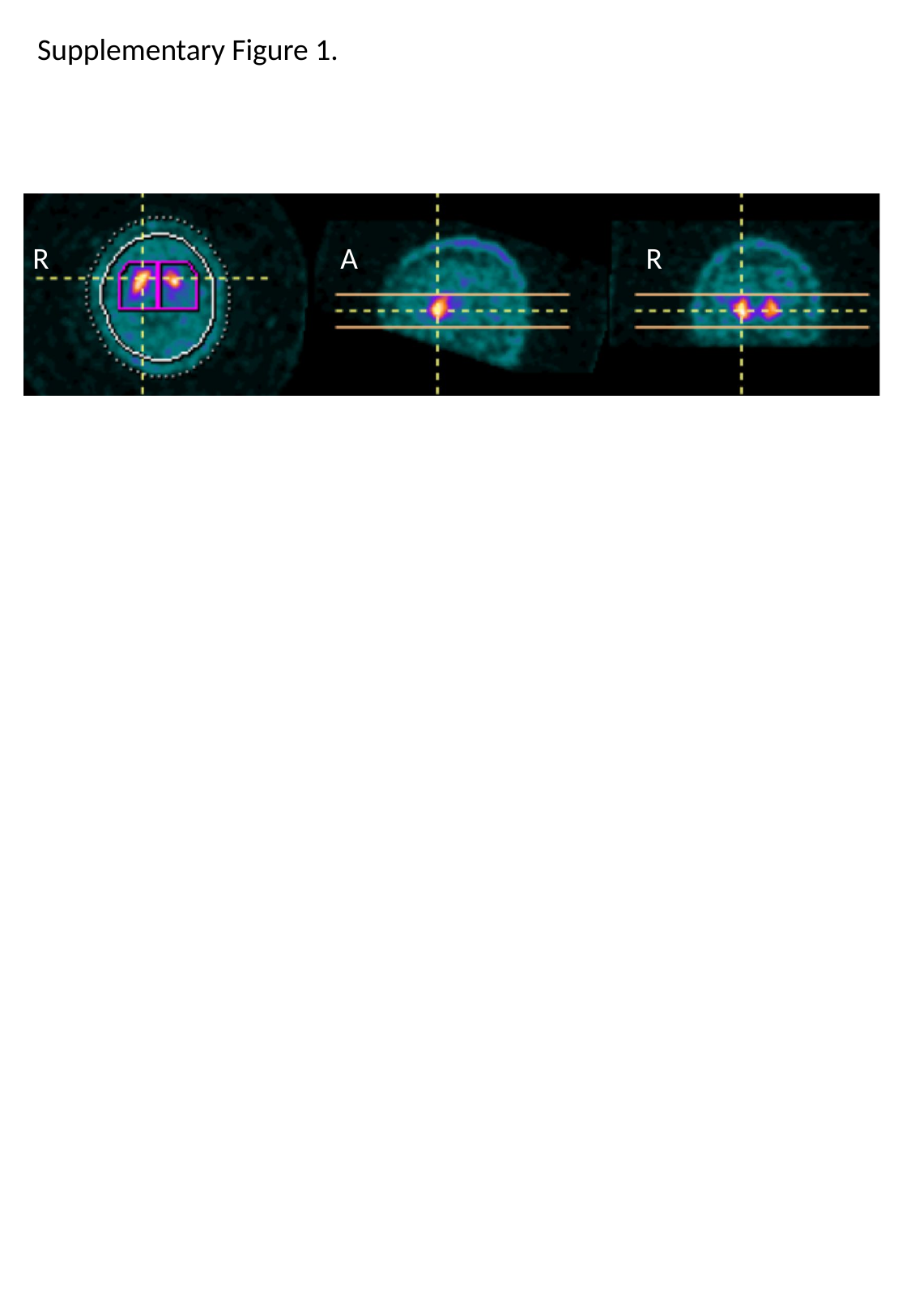

Supplementary Figure 1.
A
R
R

Supplement: Supplementary Figure 1 — A process of geometric VOI determination by the Southampton method in representative patients with dystonia. Purple pentagons represent preformed striatum ROI. The radiological technicians were allowed only to shift VOIs in the anterior–posterior and lateral directions. The yellow dotted line represents the center of the right striatum, and the yellow line represents the upper and lower limits of the striatum automatically identified by the software. The white dotted line represents the automatically determined whole brain, and the white line represents a reference ROI 20 mm inward from the white dotted line. VOI, volume of interest; ROI, region of interest. [file Presentation_1.PPTX]
